# Trends in TB case notification over fifteen years: the case notification of 25 Districts of Arsi Zone of Oromia Regional State, Central Ethiopia

**DOI:** 10.1186/1471-2458-14-304

**Published:** 2014-04-03

**Authors:** Shallo Daba Hamusse, Meaza Demissie, Bernt Lindtjørn

**Affiliations:** 1 Oromia Regional Health Bureau, Addis Ababa, Ethiopia; 2 Addis Continental Institute of Public Health, Addis Ababa, Ethiopia; 3 Centre for International Health, University of Bergen, Bergen, Norway

**Keywords:** TB, Trends, Case notification, Arsi zone, Ethiopia

## Abstract

**Background:**

The aims of tuberculosis (TB) control programme are to detect TB cases and treat them to disrupt transmission, decrease mortality and avert the emergence of drug resistance. In 1992, DOTS strategy was started in Arsi zone and since 1997 it has been fully implemented. However, its impact has not been assessed. The aim of this study was, to analyze the trends in TB case notification and make a comparison among the 25 districts of the zone.

**Methods:**

A total of 41,965 TB patients registered for treatment in the study area between 1997 and 2011 were included in the study. Data on demographic characteristics, treatment unit, year of treatment and disease category were collected for each patient from the TB Unit Registers.

**Results:**

The trends in all forms of TB and smear positive pulmonary TB (PTB+) case notification increased from 14.3 to 150 per 100,000 population, with an increment of 90.4% in fifteen years. Similarly, PTB+ case notification increased from 6.9 to 63 per 100,000 population, an increment of 89% in fifteen years. The fifteen-year average TB case notification of all forms varied from 60.2 to 636 (95% CI: 97 to 127, P<0.001) and PTB+ from 10.9 to 163 per 100,000 population (95% CI: 39 to 71, p<0.001) in the 25 districts of the zone. Rural residence (AOR, 0.23; 95% CI: 0.21 to 0.26) and districts with population ratio to DOTS sites of more than 25,000 population (AOR, 0.40; 95% CI: 0.35 to 0.46) were associated with low TB case notification. TB case notifications were significantly more common among 15-24 years of age (AOR, 1.19; 95% CI:1.03 to 1.38), PTB- (AOR, 1.46; 95% CI: 1.33 to 64) and EPTB (AOR, 1.49; 95% CI; 1.33 to 1.60) TB cases.

**Conclusions:**

The introduction and expansion of DOTS in Arsi zone has improved the overall TB case notification. However, there is inequality in TB case notification across 25 districts of the zone. Further research is, recommended on the prevalence, incidence of TB and TB treatment outcome to see the differences in TB distribution and performance of DOTS in treatment outcomes among the districts.

## Background

Despite the availability of effective treatment since the mid-1990s, TB remains a major public health problem and the second leading cause of death worldwide [1-3]. In 2011 there were about 8.7 million new TB cases and 1.4 million deaths worldwide from the disease [4]. In 1993, the World Health Organization (WHO) declared TB as a global public health emergency and recommended Directly Observed Treatment Short course (DOTS) as a standard strategy to control the disease [5,6]. DOTS aim to detect 70% of infectious cases and successfully treat 85% of them to interrupt the transmission, reduce mortality and prevent emergence of drug resistance [5,6].

In WHO Global TB report, Ethiopia ranked 7^th^ among 22 High Burden Countries and 3^rd^ in Africa in 2011 [3,7]. Moreover, TB is one of the most important infectious diseases responsible as 3^rd^ cause of hospital admission and the second top causes of death in Ethiopia [3,8]. According to the 2011 national TB survey result, the prevalence of all forms of TB was 240 and PTB + was 108 per 100,000 population [9]. However, studies from northern, southern and central Ethiopia [10-16] have shown that the prevalence of smear PTB + ranged from 76 to 189 per 100,000 population suggesting TB prevalence varied across different geographical locations of the country. Moreover, evidences form northern Ethiopia and other African counties have shown there is clustering of TB cases and variation in TB prevalence rate across different geographic settings [15,17-20].

DOTS was piloted in Ethiopia in the mid of 1992 in Arsi and Bale zones of Oromia regional state [21]. In 2010, it was gradually scaled up to the entire country and came up to have a 100% district and 90% health facility coverage [22]. Three studies, two from southern and one from northern Ethiopia, showed that the implementation of DOTS strategy over ten, eight and three years had improved the trends in TB case notification and treatment success with the expansion of DOTS programme to the general health services [5,23,24].

To our knowledge, no study has evaluated the progress of DOTS strategy in the trends of TB case notification and made comparison of its performance across districts in the country. In fact, Arsi was one of the nationally selected zones that piloted DOTS programme in 1992 [8]. However, performance of the programme including trends in TB case notification has not been assessed in the zone. The aim of this study was, therefore, to analyse trends in TB case notification over fifteen years and make comparisons among the 25 districts of Arsi zone of Oromia Regional State, Central Ethiopia.

## Methods

### Study settings

Arsi, one of the zones of Oromia Regional State, is located at 175 km southeast of Addis Ababa. It has one urban and 24 rural district with 3.1 million people residing in an area of 21,120 km^2^. It is also one of the most densely populated zones with 148 people per km^2^. About 89% of the population lives in rural areas while the remaining 11% resides in urban areas. In 2011 about 70% of zonal population lived within two-hour walking distance from a public health facility. According to the national standard universal health service coverage, population living within two-hour walking distance or 10 km radius of either a health centre or a hospital is considered to have access to DOTS and other health services [25]. Moreover, a hospital or a health centre with DOTS service during the study period was considered as a DOTS site. Accordingly, DOTS population coverage of each district of the zone was computed by taking population living within two-hour walking distance or 10 km radius (estimated at 250,000 for hospital and 25,000 for health centre) of a hospital or a health centre as nominator, and the total number of mid-year population of each district as denominator multiplied by 100.

Since 1997, the DOTS programme has been gradually expanding to the general health services of the zone. In 2011, DOTS strategies were fully integrated into all health facilities of the zone and used both as TB diagnostic and treatment units. These treatment units have standard TB Unit Registers from the National Tuberculosis and Leprosy Control Programme (TLCP).

### Study design and data collection

This was a facility-based retrospective longitudinal study design. In this case, we reviewed the profile of all forms of TB cases registered between September 1, 1997 and August 31, 2011 to analyze the trends in TB case notification and make compression of TB performance across districts in Arsi Zone of Oromia Regional State, Central Ethiopia.

All forms of TB cases registered during the study period in all health institutions that provided DOTS services in 25 districts of the zone (73 health centres and one hospital) were included in the study. TB Unit Registers in all health facilities during the period were identified by the principal investigator and brought to the regional health bureau office between January and March 2013. Then, 10 trained data clerks collected TB patients’ information on sex, age, address, TB type, patient category, date treatment started, and HIV testing and their status from the TB Unit Registers and entered the data onto a computer programme (SPSS version 20) from April to June 2013.

### Definitions of terms

Based on the National Tuberculosis and Leprosy Control Programme guideline (NLCP) adopted from WHO [8], the various types of tuberculosis (TB) are defined as follows:

Pulmonary TB smear-positive (PTB+) is a patient with at least two initial sputum smear positive for acid-fast bacilli (AFB) by direct microscopy or a patient with only one sputum smear positive for AFB and with chest radiographic abnormalities consistent with active pulmonary TB followed by clinician’s decision.

Pulmonary TB smear-negative (PTB-) is a patient with at least three initial sputum smear negative for AFB by direct microscopy and with chest radiographic abnormalities consistent with active pulmonary TB and no clinical response to two weeks of broad spectrum antibiotic therapy followed by clinician’s decision.

Extra pulmonary tuberculosis (EPTB) is tuberculosis involving organs other than the lungs, such as skin, abdomen, joints and bones, lymph nodes, pleura, genitourinary tract, and meninges. The diagnosis is based on fine needle aspiration (FNA) for histopathological examination or biochemical analysis of ascetic/pleural/cerebrospinal fluid followed by clinician’s decision to treat it with a full course of anti-TB drugs. However, a patient with three initial sputum smear negative for AFB at health centre and with no clinical response to two weeks of broad spectrum antibiotic therapy, and also suspected of EPTB at health centre were referred to hospital for further radiological and histopathological investigation before diagnosis as TB cases at health centre level.

### Measurements

Area of residence, sex, age, HIV status, type of TB, patient category and population ratio to health facilities with DOTS service [25-28] were used as independent variables, whereas TB case notification was taken as dependent variable. The independent and dependent variables were further categorized into groups for analysis.

The population size used as denominator to calculate TB case notification of 25 districts of the study area was obtained from the 1997 and 2007 National censuses [29,30]. The mid-year population of each district for each of the fifteen years was then extrapolated from the two censuses. The fifteen-year average mid-year population of each district was obtained by adding the mid-year population for each of the fifteen years (1997 to 2011) in each district and then dividing it by 15. The fifteen-year average of all forms of TB case for each district was also computed by adding all forms of TB case notified by each district in each year (between 1997 and 2011) and dividing it by 15. The same procedure was followed to compute the fifteen-year average of PTB + case for each district. Finally, the 15-year average of all forms of TB case notification and PTB + case notification for each district was computed by dividing the 15-year-average of all forms of TB and PTB + cases notified in each district by the 15-year-average mid-year population of their respective district and multiplying it by 100,000 population.

$$\begin{array}{l} \mathrm{Fifteen}‒\mathrm{year}\;\mathrm{ATBCN}\\ =\left(\frac{15‒\mathrm{year}\;\mathrm{average}\;\mathrm{of}\;\mathrm{all}\;\mathrm{TB}\;\mathrm{case}\;\mathrm{notified}\;\mathrm{in}\;\mathrm{each}\;\mathrm{district}}{15‒\mathrm{year}\;\mathrm{average}\;\mathrm{mid}\;\mathrm{year}\;\mathrm{population}\;\mathrm{of}\;\mathrm{their}\;\mathrm{respective}\;\mathrm{districts}}\right)\\ \;\times \;100,000\;\mathrm{population} \end{array}$$

### NB: ATBCN is fifteen-year average of TB case notification

The PTB + case detection rate (CDR) for each year (1997 to 2011) of the zone was computed as follows: first we calculated the total number of expected incidence of PTB + cases for each mid-year population of the zone (104/100,000 for Oromia) based on the 2011 National TB Prevalence survey result where Arsi zone was one of the zones in the region [9]. Then we calculated the PTB + CDR by taking the total number of PTB + cases notified in each year in the zone as nominotor and the total expected PTB + incidence cases of the zone for each year as denomintor and multiplying it by 100.

$$\mathrm{PTB}\;+\;\mathrm{CDR}\;\mathrm{for}\;\mathrm{each}\;\mathrm{year}\;\left(1997\;\mathrm{to}\;2011\right)\;=\;\frac{\mathrm{Total}\;\mathrm{number}\;\mathrm{of}\;\mathrm{PTB}\;+\;\mathrm{case}\;\mathrm{notified}\;\mathrm{in}\;\mathrm{each}\;\mathrm{year}\;\mathrm{in}\;\mathrm{the}\;\mathrm{zone}}{\mathrm{Total}\;\mathrm{number}\;\mathrm{of}\;\mathrm{expected}\;\mathrm{PTB}\;+\;\mathrm{case}\;\mathrm{in}\;\mathrm{each}\;\mathrm{year}\;\mathrm{in}\;\mathrm{the}\;\mathrm{zone}}\times 100$$

Since 2008, all TB patients have been offered provide initiated voluntary counselling and testing service for HIV. Hence, TB-HIV co-infection rate for each district was computed by taking all TB patients tested for HIV and found to be HIV positive as nominator and all TB patients tested for HIV during the study period as denominator multiplied by 100.

### Statistical analysis

Data were coded and double entered by trained data clerks using Epi-info statistical software version 7. Later, they were exported to IBM SPSS version 20 for data checking, cleaning, and bivariate and multiple logistic regression analysis. Descriptive analyses such as frequency, mean, and standard deviation were computed as appropriate. Adjusted odds ratio was used to determine the strength of association between the study variables at 95% CI and P value <0.05. The model adequacy and co-linearity assumptions were checked to be satisfied based on appropriate methods designed for the study.

In the bivariate and multivariate binary logistic regression analyses of TB case notification, we excluded Assela town (urban district) from the analysis of fifteen-year average of all forms of TB case notification of districts in the zone. This was because Assela Referral Hospital in Assela town was used as referral center for TB cases from the entire zonal population and the neighboring zones. Therefore, if we take the population of the town as a denominator to calculate its TB case notification, we might overestimate the TB case notification of the zone and the town. Consequently, after excluding Assela town form the analysis, the fifteen year-average of all forms of TB case notification of the 24 rural districts of the zone was found to be 120/100,000 population and the study subjects were normally distributed. Therefore, we took this as cut-off value in the dichotomization of all forms of TB case notification into below 120 and above 120 per 100,000 in the bivariate and multivariate binary logistic regression analyses.

### Ethical approval

Ethical approval was obtained from institutional Review Board of Oromia Regional State Health Bureau, Ethiopia. Formal permission to use the data was obtained from the officials.

## Results

### General characteristics of the study subjects

A total of 41,965 TB patients were registered in 74 treatment units of Arsi zone between September 1, 1997 and August 31, 2011. Gender wise, more than half, 22,743 (54.2%), were males and residence wise, slightly over a third, 14,052 (36%) were urban residents. The age of the patients ranges from one to 98 years with mean age of 28.7 years (SD +15.3 years). The majority (93%) were new and over a third of them, 15,370 (36%) were pulmonary smear-positive; 15,102 (36%) were pulmonary smear negative; and 11,447 (27.3%) were extra-pulmonary tuberculosis cases (Table 1).

**Table 1:** General characteristics of the study subjects (n = 41,965), Arsi Zone, Oromia Regional State, Central Ethiopia, 1997-2011

**Patient Characteristics**	**Number**	**Percentage**
**Age Category**		
0-14 years	5,587	13.3
>14 years	36,004	85.8
Unknown	374	0.9
**Sex**		
Male	22,743	54.3
Female	18,815	44.8
Unknown	382	0.9
**Patient Category**		
New	39,010	93.0
Relapse	804	1.9
Failure	61	0.1
Defaulter	99	0.2
Transfer-In	894	2.1
Other Cases	705	1.7
Unknown	392	0.9
**TB Classification**		
Pulmonary/Positive	15,370	36.6
Pulmonary/Negative	15,102	36.0
Extra-pulmonary	11,447	27.3
Missing (unknown)	46	0.1

### The Trends in DOTS Site Expansion and TB Case Notification

**Figure 1:**
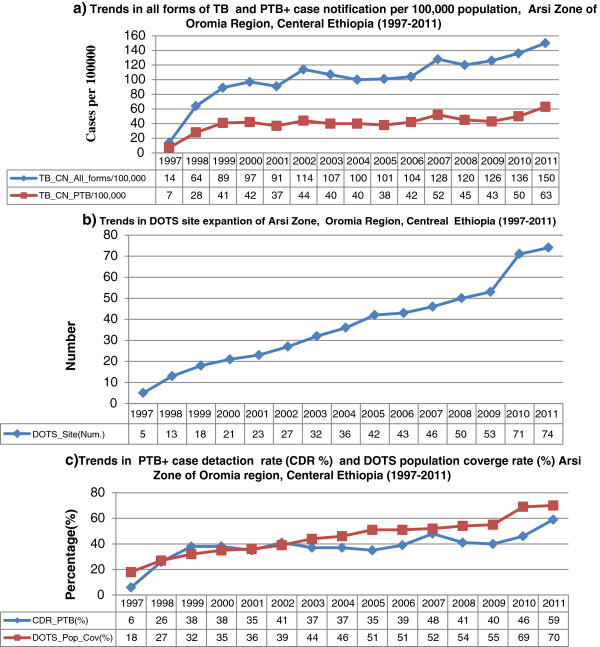
**Trends in all forms of TB, Pulmonary smear positive TB case notification, DOTS sites expansion and DOTS population coverage, Arsi Zone of Oromia Regional State, Central Ethiopia, 1997-2011.** N.B: Trends in TB case notification increased with DOTS sites expansion for all forms of TB $X_{\mathrm{trend}}^{2}$ =75.2, p<0.001, PTB+ $X_{\mathrm{trends}}^{2}$ =27.74, P <0.001) and CDR PTB+ $X_{\mathrm{trend}}^{2}$ =26.794, p<0.001.

The trend in TB case notification was significantly associated with the number of DOTS sites in the zone (Figure 1a and 1b, $X_{\mathrm{trend}}^{2}$ = *75.2*, *P* < 0.001) which gradually increased from five in 1997 to 23 in 2001 and from 46 in 2007 to 74 in 2011 (Figure 1b). Similarly, the DOTS population coverage increased with the number of DOTS sites. It went from 18% in 1997 to 36% in 2001 and from 52% in 2007 to 70% in 2011 (Figure 1c). Subsequently, with increased number of DOTS sites, all forms of TB case notification of the zone increased from 14.3 in 1997 to 150 per 100,000 population in 2011, an increment of 91% in fifteen years. Figure 1a also shows there was a steady upward trend in all forms of TB case notification in the first four years of DOTS introduction (from 14.3 in 1997 to 96.5 per 100,000 in 2000). However, this rising trend has been stabilized in the range between 91.2 and 104 per 100,000 population between the years 2001 and 2006. The trends further increased again to 128 in 2007 and to 150 per 100,000 population in 2011.

Likewise, PTB + case notification of the zone increased from 7 in 1997 to 63 per 100 000 population in 2011 with an increase of 89% in fifteen years. Similarly, the trend in TB case detection rate (CDR) of the zone went up from 6.4% in 1997 to 34.5% in 2001, and from 48.3% in 2007 to 58.7% in 2011 ($X_{\mathrm{trend}}^{2}$ = *26.8*, *P* <*0.001*) (Figure 1c). Similar to the trend in all forms of TB case notification, PTB + in the first four years of DOTS introduction increased from 6.9 in 1997 to 41 per 100,000 population in 2000. However, through the years 2001 to 2010, with the exception of 2007, the trend stabilized in the range between 35 and 46 per 100,000 population (Figure 1a).

### Fifteen-year average TB case notification by districts

**Figure 2:**
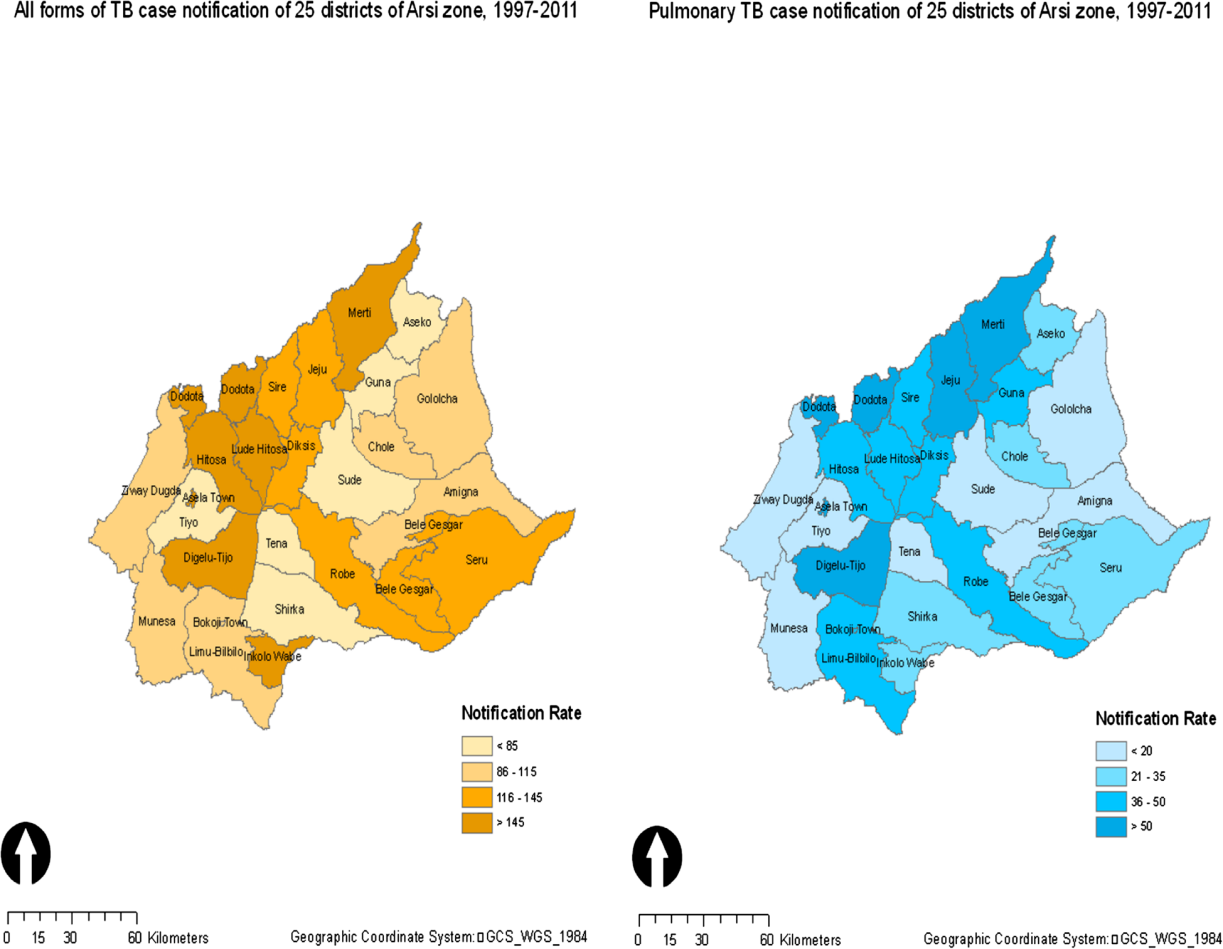
Fifteen-year average of all forms and PTB+ve TB case notification of 25 districts of Arsi Zone, 1997-2011.

Table 2 shows that fifteen-year average TB case notification across the 25 districts of the zone was unevenly distributed. All forms of TB notification were high (over 145 per 100,000 population) for seven districts and low (below 85 per 100,000) for six districts (Figure 2). The highest all forms of TB case notification was from Assela town (636.1 per 100,000) followed by Dodota district (314.2 per 100,000) (Table 2). Similarly, TB and HIV co-infection in Assela town and Dodota district was 24.2% and 16.4% respectively, high compared to other districts. Nevertheless, the lowest (63 per 100,000) all forms of TB case notification was observed in Tiyo and Aseko where TB and HIV co-infection was 6.9% and 2% respectively. This is less than 9.4% of the zonal average TB and HIV co-infection (Table 2).

**Table 2:** **Yearly average TB case notification**/**100**,**000 population and TB**/**HIV co**-**infection rate** (%), **25 districts in Arsi Zone of Oromia region**, **Centeral Ethiopia** (**1997**-**2011**)

**Name of district**	**Average fifteen-year mid-year population**	**No. of TB cases notified (Avg.)**		**TB case notification/100,000**		**TB-HIV co-infection**	
		**All forms**	**PTB+ve**	**All forms**	**PTB+**	**Number of tested**	**HIV positive (%)**
Tiyo	80,384	50	9	62.5	10.9	130	9 (6.9%)
Amigna	67,862	61	11	90.3	16.3	178	18 (9.6%)
Aseko	77,930	49	23	63.2	29.6	304	6 (2%)
Assela town	62,325	396	102	636.1	163	1415	342 (24.2%)
Bele/Ge	68,517	84	18	122.9	25.7	167	6 (3.6%)
Cholle	82,728	71	27	85.4	33	282	30 (10.6%)
Digalu/Tijo	130,142	207	70	159	53.7	838	62 (7.4%)
Diksis	66,987	93	26	138.8	38.1	224	5 (2.2%)
Dodota	59,583	187	90	314.2	150.4	444	73 (16.4%)
Gololcha	159,521	139	28	86.8	17.5	491	7 (1.4%)
Guna	70,752	58	30	81.3	42.2	240	12 (5%)
Hetosa	115,089	187	56	162.9	48.4	688	63 (9.2%)
Honkolo/Wa	54,257	79	11	145.1	20.2	122	3 (2.5%)
Jaju	114,972	155	59	135.2	51.1	788	34 (4.3%)
Limu/Bibi	167,414	146	63	87	37.8	429	46 (10.7%)
Lode Hitosa	99,259	146	43	146.7	43	433	61 (14.1%)
Merti	83,763	143	42	171.1	50.1	595	56 (9.4%)
Munessa	154,299	165	23	106.7	14.9	423	42 (9.9%)
Robe	153,067	205	68	133.8	44.4	517	30 (5.8%)
Shirka	151,782	116	37	76.5	24.6	211	7 (3.3%)
Sire	68,533	98	33	142.4	48.1	395	27 (6.8%)
Sude	136,904	112	27	82	19.9	293	3 (1%)
Tena	61,337	37	10	68.8	16.4	206	14 (6.8%)
Z/Dugda	111,979	128	20	87.2	17.9	412	16 (3.9%)
Seru	44,406	52.2	11	82.5	40.5	203	12 (5.9%)

Similarly, the 15-year-average of PTB + case notification for the 25 districts of the zone was 42.3 per 100,000 population. Table 2 and Figure 2 show that the fifteen-year average PTB + case notification for five districts was high and above 50 per 100,000 population compared to other seven districts with case notification of less than 20 per 100,000 population. As observed in all forms of TB, the highest fifteen-year average PTB + case notification was observed in Assela town (163 per 100,000) followed by Dodota district (150.2 per 100,000 population). The lowest was from Tiyo (10.9 per 100,000) preceded by Munessa (14.9 per 100,000 population) (Table 2).

In binary logistic regression analyses, area of residence, age, type of TB and population ratio to DOTS sites of the districts were associated with low TB case notification. In the final model, rural residence (AOR, 0.23; 95% CI: 0.21 to 0.26) and districts with population ratio to DOTS sites with more than 25,000 population (AOR, 0.40; 95% CI: 0.35 to 0.46) were associated with low TB case notification. However, TB case notifications were significantly more common among 15-24 years of age (AOR, 1.19; 95% CI: 1.03 to 1.38), PTB- (AOR, 1.46; 95% CI: 1.33 to 64) and EPTB (AOR, 1.49; 95% CI; 1.33 to 1.60) TB cases (Table 3).

**Table 3:** Factors associated with case notification rate among patients registered from 1997-2011 in Arsi Zone, Oromia Regional State, Central Ethiopia

**Variables**	**Category**	**Category of case notification**	**COR (95% CI)**	**AOR (95% CI)**	
		**<120 cases/100,000**	**>120 cases/100,000**		
Residence (N = 39,471)	Urban	2,554 (17.5)	11,598 (82.5)	1.00	1.00
	Rural	9,310 (36.8)	16,009 (63.2)	0.36 (0.35,0.38)**	0.23 (0.21,0.26)**
Sex (N = 41,583)	Male	6,876 (30.2)	15,892 (69.8)	1.00	1.00
	Female	5,716 (30.4)	13,099 (69.6)	0.99 (0.95,1.03)	0.99 (0.89,1.06)
Age (N = 41,591)	0-14 years	1,676 (30.0)	3,911 (70.0)	1.00	1.00
	15-24 years	4,059 (31.2)	8,942 (68.8)	0.94 (0.88,1.01)	1.19 (1.03,1.38)*
	25-49 years	5,387(29.7)	12,729 (70.3)	1.01(0.95,1.08)	1.10 (0.96,1.28)
	≥50 years	1,515 (31.0)	3,372 (69.0)	0.95 (0.88,1.04)	1.09 (0.92,1.39)
TB Type (41,919)	PTB+	5,226 (34.0)	10,144(66.0)	1.00	1.00
	PTB -	3,904 (25.9)	11,198 (74.1)	1.48 (1.41,1.55)**	1.46 (1.33,1.64)**
Population ratio to DOTS sites in the districts (N = 41,965)	EPTB	3,634 (31.7)	7,813 (68.3)	1.11 (1.05,1.17)**	1.49 (1.33,1.6)**
	≤ 25,000	1,112 (16.3)	5,713 (83.7)	1.00	1.00
	25,001-40,000	8,280 (43.6)	10,713 (56.4)	0.25 (0.24,0.27)**	0.41 (0.36,0.47)**
		3,400 (21.1)	12,747 (78.9)	0.73 (0.68,0.79)**	0.40 (0.35,0.46)**

NB: - * Significant at P-value<0.05 and ** Significant at P-value<0.001.

With the national standard that one health centre with DOTS service can be accessible for 25,000 in TB care, the ratio of population size to the number of DOTS sites was calculated as total number of population in the district divided by number of DOTS sites in the same district. (Total number of population in the District/Total number DOTS sites in the same district).

## Discussion

The study has confirmed that the expansion of DOTS strategy led to improved TB case notification across 25 districts of Arsi zone. However, it was identified that there is inequality and uneven distribution in TB case notification among the districts. The new knowledge about difference in TB case notification across districts is an important indication calling for investigation to identify if this difference is related to difference in the prevalence of the disease or to the existence of undetected TB patients among districts with low TB case notification.

These findings are significant particularly in resource-constrained settings where there is limited health infrastructure and inadequate physical and financial access across different geographical settings. If this inequality in TB case notification resulted from access to health care, then it may limit the effectiveness of DOTS strategy to attain the global WHO 70% TB case detection rate and 85% treatment success rate aimed at interrupting TB transmission, reducing mortality and preventing emergence of drug resistance [5,31].

The DOTS strategy was initially introduced in 1992 as a national pilot in a health centre and a hospital in Arsi zone [8,21] followed by systematic scaling up of the control programme to other health facilities. This stepwise DOTS expansion started in health facilities at the district capital and then continued to sub-district level health facilities. Meanwhile, with the exception of health facilities under construction, full DOTS service coverage was achieved in eight years. This goes in line with previous reports from Southern Ethiopia where 73% of zonal [23] and full zonal and districts [5] DOTS service coverage were achieved in seven years following the introduction of DOTS strategy.

The continuous DOTS-site expansion in Arsi zone over the past fifteen years might be due to high political commitment in securing necessary resource for the establishment of laboratory network for effective TB diagnosis, treatment, and monitoring, and in ensuring an uninterrupted supply of anti-TB drugs [5,32].

Consequently, between 1997 and 2011, TB case notification increased from 14 to 150 for all forms and from 6.9 to 63 per 100,000 population for PTB+. This finding substantiates results of previous studies in Southern Ethiopia where the ten-year trend in all forms of TB case notification increased from 45 to 143 [5] and PTB + from 49 to 126 per 100,000 population in eight years [23]. However, our study showed that under the current passive TB case findings, the trend in TB case notification did not persistently increase though out the study period. This might warrant the involvement of health extension workers in active TB case finding in the Ethiopian context so as to achieve the MDG of 70% of TB case detection rate [33].

The case detection rate (CDR), estimated by the proportion of PTB + cases notified form the total expected PTB + incidence cases in the community, showed an increase from 7% in 1997 to 63% in 2011. Previous studies [5,23] also indicated that the trends in PTB + CDR more than doubled in eight years. Likewise, in Vietnam the CDR increased by more than six-fold in fifteen years [32]. The most likely explanation for the increase in CDR over time could be due to the real increase in PTB + case detection rate following decentralization of DOTS strategy led to improved access to laboratory service.

The other reason could be the improvement in recoding and reporting of detected TB cases following the introduction of DOTS without real increase of TB case detection rate [34]. Nevertheless, the increased trend might also be due to true increase in TB incidence cases fueled by the powerful interaction between HIV and tuberculosis [35]. The TB and HIV co-infection among tested TB patients in our study was 9.4%. It might also be due to the notification of large backlog of TB cases that resulted from improved TB diagnostic access [34].

The upward trend in CDR in this study was slightly higher than report from Southern Ethiopia [5] but lower than other reports from the same region and Vietnam [23,32]. The explanation for this discrepancy might be due to variation in DOTS performance across different study areas. The difference could also be due to the variation in disease burden across different geographical settings [15,17-20].

The PTB + case notification and CDR of the zone steadily increased during the first six years of DOTS implementation. However, despite a notable increase in the number of TB diagnostic centres, the PTB + case notification and CDR seem to be stable during the years 2002–2010. The result corroborates the previous report that indicated the number of reported TB cases did not proportionally increase with the number of TB DOTS sites after five years of DOTS introduction [5]. Moreover, the finding confirms pervious study reports where an increase in the trends of PTB + case detection rate in the first five years [5,23] and seven years [32] of DOTS introduction was followed by stabilizing of case detection rates. The expansion of DOTS-sites may help to improve case notification to a certain point while increase in coverage may contribute to marginal increment in case finding unless other community level interventions are introduced [33].

In the mathematical model used to predict the WHO target of 70% TB case detection rate and 85% cure rate in countries where the incidence of tuberculosis is stable and HIV-1 absent, there would be a reduction of TB incidence rate by 11% and death rate due to TB by 12% per year [36]. However, in this study where there is low PTB + CDR (63%) and high prevalence rate of HIV among TB patients (9.4%), it is not convincing to argue that the decline in TB incidence led to stability in PTB + case detection rate after six years of DOTS implementation.

In this study, we identified variations in TB case notifications among 24 rural districts and one urban district of Arsi zone. The findings show that TB case notification of Dodota district among the 24 rural districts was very high compared to Tiyo, Amigna, Shirka, Sude and Tena districts. Such a situation in Dodota district might be explained by the true increase in TB incidence cases fueled by the powerful interaction between HIV and tuberculosis [35] as TB and HIV co-infection of the district is very high (16.4%) compared to 9.4% of zonal average.

The overall fifteen-year average PTB + case notification of the districts was 42.3 per 100,000 population with a fourteen fold variation between different districts (10.9 to 150 per 100,000 in rural and 166 per 100,000 in urban areas). This dissimilarity in PTB + case notification among districts may be an indication of inequity in TB case findings or heterogeneity in TB incidences across 25 districts [15,17,18,37] caused by diversity in TB risk factors [32,38]. This difference could be a result of the defect in the current passive facility-based TB case findings of DOTS policy in uniformly notifying TB cases across districts either due to limited ability of the health system to detect TB cases or poor health care seeking behaviour particularly for those with TB [20].

In this study factors like area of residence, age of patients, type of TB and the ratio of population size to DOTS sites were found to be associated with the level of TB case notification. This is in agreement with studies conducted elsewhere [26-28] where TB case notification was associated with urban residence, age of patient, access in TB care and type of TB.

Although our study demonstrated the usefulness of facility-based data analysis at district level, it has some limitations. This retrospective facility-based longitudinal study has no socio-economic and environmental data which is the inherent limitation of retrospective study. Therefore, the absence of socio-economic and environmental data in this study could affect the result as it might have an association with the variation in TB case notification. There might also be bias in TB case notification of the districts due to the extrapolation of census data from 1997 and 2007 to get up-to-date denominator of the population. However, there might be unevenly distributed population growth rate across years of the study period and districts. If this were the case, there would be either under or overestimation of TB case notification. The notification in some districts might be overestimated due to existence of some health facilities with good history of TB care that could attract more patients from nearby districts.

### Conclusions

The introduction and expansion of DOTS in Arsi zone has improved the overall TB case notification. However, there is inequality across the 25 districts of the zone. Universal achievement of the global WHO target of 70% case detection rate across different geographical settings with diversity in socio-economic condition will continue to be a challenge. This study demonstrated to public health professionals the importance of using existing health facilities data in providing necessary information about TB case notification across different districts. Thus, it is important to enable the concerned bodies to try out more locally applicable and effective strategies to attain MDG in TB control. To this end, further research is recommended on the prevalence and incidence of tuberculosis and also on TB treatment outcome to see the differences in the distribution of the disease and performance of DOTS strategy in treatment outcomes across the 25 districts.

## Competing interests

The author’s declare that they have no competing interests.

## Authors’ contributions

SDH was the principal investigator who participated in designing and conducting the study. Further, he was involved in analyzing the data and writing the manuscript. BL took part in the design of the study, analysis of data and write up of the manuscript and MD participated in designing the study and writing the manuscript. All authors read and approved the final manuscript.

## Pre-publication history

The pre-publication history for this paper can be accessed here:

http://www.biomedcentral.com/1471-2458/14/304/prepub
